# Crosstalk of heat shock proteins and antioxidants with peroxisome biogenesis supports wheat thermotolerance

**DOI:** 10.1038/s41598-026-48451-0

**Published:** 2026-05-10

**Authors:** J. E. Shenoda, Marwa N. M. E. Sanad, Aida A. Rizkalla, Mona H. Hussein, S. El-Assal

**Affiliations:** 1 Genetics and Cytology Department, Biotechnology Research Institute, National Research Centre (NRC), Cairo, Egypt; 2 Genetics Department, Faculty of Agriculture, Cairo University, Giza, Egypt

**Keywords:** Wheat thermotolerance, Gene expression, Peroxisome biogenesis, Heat shock proteins (HSPs), Antioxidant enzymes, Phenotype-gene association analyses, Genetics, Molecular biology, Plant sciences, Climate sciences

## Abstract

Rising temperatures globally impose coupled proteotoxic and oxidative stresses that limit spring wheat (*Triticum aestivum*) productivity, yet how protein quality control, redox homeostasis, and peroxisome dynamics are coordinately regulated during thermotolerance remains unclear. Here, we investigated the integrated roles of heat shock proteins (HSPs), antioxidant defenses, and peroxisome biogenesis in two contrasting wheat genotypes differing in heat stress tolerance at anthesis**:** Misr2 (tolerant) and Line4 (susceptible). Heat responses were characterized by osmoprotectant accumulation (total soluble sugars, proline), oxidative stress markers (malondialdehyde, hydrogen peroxide), antioxidant enzyme activities (CAT, SOD, POX), peroxisome abundance, and expression of stress-responsive genes. Integrated phenotype–gene association analyses distinguished thermotolerant and susceptible responses in Misr2 and Line4 by resolving coordinated regulatory modules. Expression profiles of eight candidate genes (*TaHSP70, TaHSP90, TaSOD, TaCAT1, TaPEX11.3, TaPEX11.4, TaFIS1A, and TaDRP5B*), analyzed in conjunction with phenotypic traits, revealed modular gene networks linking antioxidant defenses, HSP-mediated proteostasis, and peroxisome proliferation. *TaHSP70, TaPEX11.4, and TaCAT1* functioned as regulatory hubs, *TaDRP5B* and *TaSOD* as phenotypic switch nodes, and *TaPEX11.3, TaFIS1A*, and *TaHSP90* as context-dependent responders. Together, these findings uncover modular crosstalk among heat-stress pathways that underpin wheat thermotolerance, providing molecular targets for breeding heat-resilient cultivars under climate change.

**Supplementary Information:**

The online version contains supplementary material available at 10.1038/s41598-026-48451-0.

## Introduction

Heat stress ranks among the most serious abiotic constraints limiting global wheat (*Triticum aestivum*) productivity, and climate change is expected to intensify this threat by increasing the frequency, duration, and severity of heatwaves^1^. Wheat yields are particularly sensitive to temperature increases, with production projected to decline by approximately 6% for every 1 °C rise in average seasonal temperature^2^. In field-grown wheat, exposure to high temperatures during anthesis and grain filling can reduce yields by up to 40%^3,4^.

In arid and semi-arid regions such as Egypt, heat-induced yield penalties are exacerbated by high baseline temperatures and frequent heat episodes during critical reproductive stages, constraining domestic wheat productivity. As a result, despite sustained cultivation efforts, Egypt currently imports more than half of its wheat demand, making wheat a strategic food security–critical crop (FAOSTAT)^5^. Reducing yield losses caused by heat stress is therefore a key prerequisite for narrowing the production–consumption gap. Addressing this challenge requires identifying and breeding heat-tolerant wheat genotypes that maintain productivity under rising temperatures.

At the cellular level, heat stress disrupts homeostasis by triggering the overproduction of reactive oxygen species (ROS), which leads to oxidative damage in organelles such as chloroplasts and mitochondria^6^. While ROS act as essential signaling molecules at moderate levels, their excessive accumulation causes lipid peroxidation, chlorophyll degradation, damage to thylakoid membranes, and impaired photosynthesis^7–11^. Plants mitigate these effects by activating a range of protective mechanisms, including the accumulation of osmoprotectants (e.g., proline and soluble sugars)^11–13^, the expression of heat shock proteins (HSPs)^14^, adjustments in carbon allocation and soluble sugar metabolism^15^ activation of stress-related signaling pathways, and enhancement of antioxidative systems such as superoxide dismutase (SOD), catalase (CAT), and peroxidase (POX)^16,17^. Notably, heat-induced antioxidant and biochemical responses have been shown to vary across wheat developmental stages, including anthesis and grain filling^18–21^.

Heat shock proteins, including HSP70 and HSP90, act as molecular chaperones that prevent protein misfolding and aggregation at elevated temperatures^22,23^. Cells tightly regulate the expression of genes that promote the synthesis of heat shock proteins through temperature sensing, signal transduction, and the binding of heat shock transcription factors to specific DNA elements^24^. Although the roles of these protective components—including antioxidant defenses, heat shock proteins, and osmoprotectant accumulation—are well documented individually under abiotic stress conditions^8,13,14^, the regulatory coordination and potential crosstalk among these pathways remain poorly understood, particularly under field heat stress conditions.

In addition to the well-characterized roles of antioxidant enzymes and heat shock proteins, peroxisomes have recently gained attention for their involvement in maintaining cellular redox balance under abiotic stress conditions^25,26^. These organelles play a central role in the generation and detoxification of ROS, particularly hydrogen peroxide (H₂O₂), making them integral to the plant’s oxidative stress response^27,28^. Biochemical quantification of peroxisome abundance is a potential biomarker of plant responses to abiotic stress and stress tolerance, as previously demonstrated in several studies^29–31^. The ability of peroxisomes to proliferate in response to environmental stimuli highlights their metabolic plasticity and adaptability toward fluctuating redox demands and enhanced ROS detoxification under stress conditions^26–28^. Despite this, the regulatory coordination between peroxisome biogenesis and other heat-responsive pathways, such as antioxidant defense and HSP expression, remains largely unexplored, particularly in wheat, especially under field conditions.

Recent studies have further emphasized the importance of genotype-specific mechanisms in heat stress tolerance. For example, Pratap et al.^32^ investigated the mechanisms underlying heat stress tolerance by comparing physiological responses, yield performance, and proteome profiles of two heat-susceptible and two heat-tolerant wheat cultivars. Their findings revealed apparent differences in regulatory networks, stress protein abundance, and heat-responsive signaling pathways between the contrasting genotypes. Similarly, in wheat (*Triticum aestivum*),^12^ reported marked upregulation of heat-responsive genes and accumulation of biochemical markers associated with terminal heat stress tolerance.

We hypothesize that heat tolerance in wheat involves coordinated mechanisms that limit cellular damage and regulate reactive ROS homeostasis. Accordingly, genes associated with these protective processes are expected to exhibit regulatory crosstalk, with network organization differing between heat-tolerant and susceptible genotypes. Based on this premise, we focused our investigation on potential interactions among genes involved in peroxisome biogenesis, antioxidant activity, and heat shock protein expression to uncover regulatory mechanisms underlying thermotolerance. This study addresses critical gaps in understanding genotype-specific responses to heat stress by identifying distinct molecular networks in tolerant versus susceptible wheat genotypes. Together, these findings provide insights that can inform future breeding strategies for developing heat-resilient cultivars.

## Results

### Grain yield in response to heat stress

**Fig. 1:**
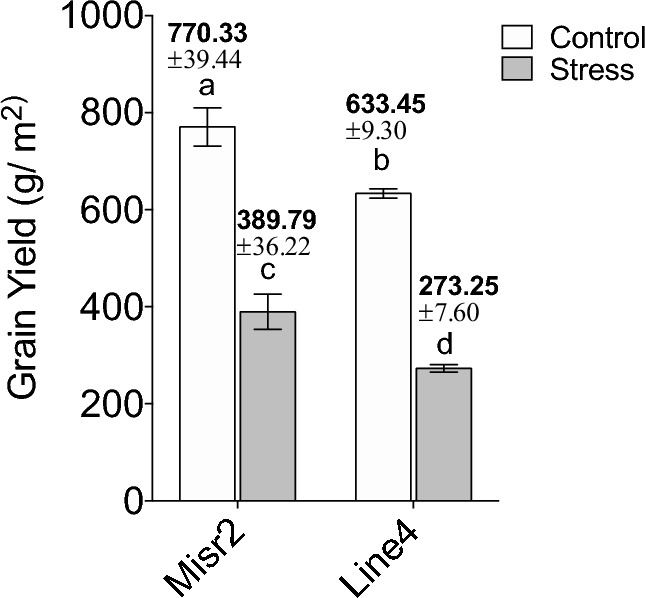
Grain yield (g/m2) of two wheat genotypes, Misr2 (tolerant) and Line4 (susceptible), under control and heat stress conditions. Heat stress induced by late sowing resulted in significant reductions in grain yield for both genotypes. Misr2 maintained higher productivity (389.79 ± 36.22 g/m2) compared to Line4 (273.25 ± 7.60 g/m2) under stress. Values represent means ± standard deviation from three biological replicates. Different letters (a, b, c, d) indicate statistically significant differences (p < 0.05) as determined by [e.g., ANOVA with Tukey’s test].

Delaying the sowing date by 57 days increased the seasonal minimum and maximum temperatures by 1.89 and 3.44 °C, respectively (See Figure S1). Heat-stressed plants experienced higher temperatures from the onset of anther development until sampling compared with non-stressed plants. Under control conditions, the grain yield of Misr2 was 770.33 ± 39.44 g/m^2^, while Line4‘s grain yield was 633.45 ± 9.30 g/m^2^ (Fig. 1). Heat stress reduced grain yield by 49% in Misr2 (389.79 ± 36.22 g/m^2^) and by 57% in Line4 (273.25 ± 7.60 g/m^2^), confirming Misr2’s greater resilience to heat stress.

### Genotype-specific physiological responses to heat stress

**Fig. 2:**
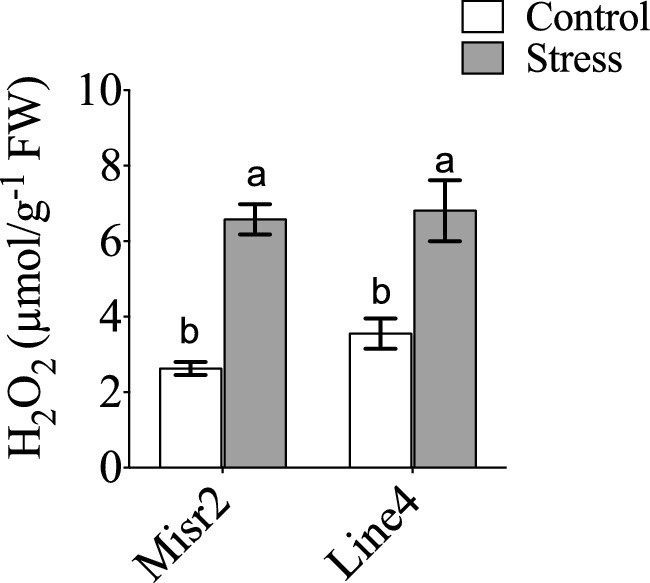
Effect of heat stress on hydrogen peroxide content (H_2_O_2_) in the tolerant (Misr2) and susceptible (Line4) wheat genotypes grown under control and stress environments. Vertical bars with different lowercase letters above are significantly different between treatments (per genotype) at *P* = *0.05* by Tukey’s test, capped bars represent SD.

**Fig. 3:**
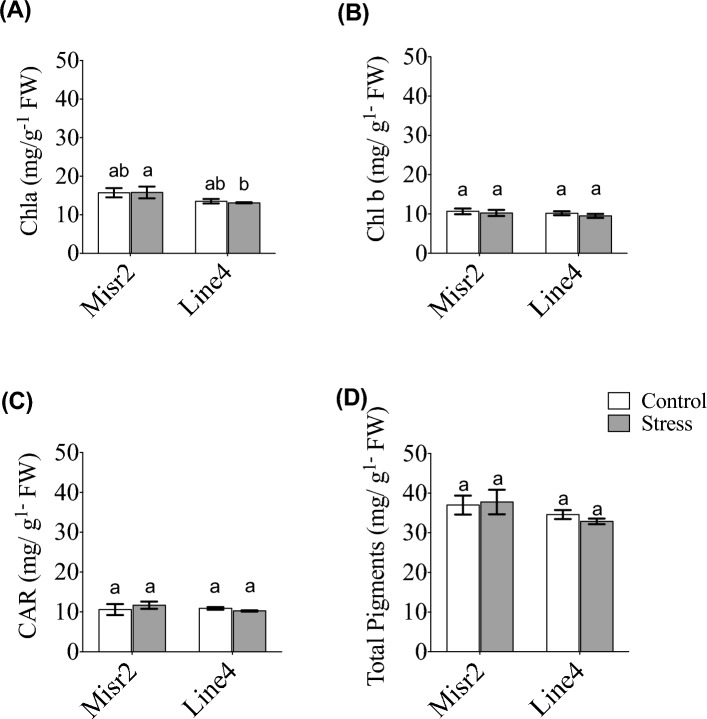
Effect of heat stress on (**A**) chlorophyll a (*Chl a*), (**B**) chlorophyll b (*Chl b*), (**C**) total carotenoids (CAR), and (**D**) total pigments in the tolerant (Misr2) and susceptible (Line4) wheat genotypes grown under control and stress environments. Vertical bars with different lowercase letters above are significantly different between treatments (per genotype) at *P* = *0.05* by Tukey’s test, capped bars represent SD.

Heat stress markedly increased H₂O₂ levels in both genotypes compared with normal sowing, indicating enhanced ROS accumulation (Fig. 2). However, neither genotype, treatment, nor their interaction significantly affected chlorophyll a, chlorophyll b, carotenoids, or total pigment content (Fig. 3A–D), suggesting both genotypes maintained photosynthetic pigment stability under heat stress.

**Fig. 4:**
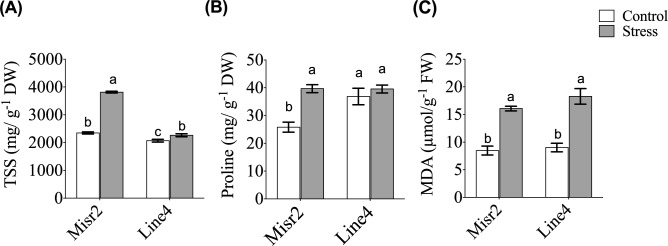
Effect of heat stress on (**A**) total soluble sugars (TSS) , (**B**) proline content , and (**C**) malondialdehyde (MDA) in the tolerant (Misr2) and susceptible (Line4) wheat genotypes grown under control and stress environments. Vertical bars with different lowercase letters above are significantly different between treatments (per genotype) at *P* = *0.05* by Tukey’s test, capped bars represent SD.

In terms of biochemical adjustments, heat stress elevated total soluble sugars (TSS) and malondialdehyde (MDA) levels in both genotypes (Fig. 4A,C). Although the two genotypes did not differ significantly in proline content under stress, Misr2 accumulated more proline than Line4 relative to their respective control levels (Fig. 4B). Misr2 also maintained higher TSS levels than Line4 under heat stress (Fig. 4A). These results indicate that TSS and proline may contribute to Misr2’s superior heat tolerance.

**Fig. 5:**
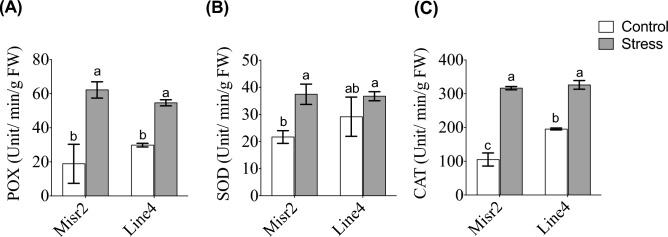
Effect of heat stress on (**A**) peroxidase (POX), (**B**) superoxide dismutase (SOD), and (**C**) Catalase (CAT) activities, in the tolerant (Misr2) and susceptible (Line4) wheat genotypes grown under control and stress environments. Vertical bars with different lowercase letters above are significantly different between treatments (per genotype) at *P* = *0.05* by Tukey’s test, capped bars represent SD.

**Fig. 6:**
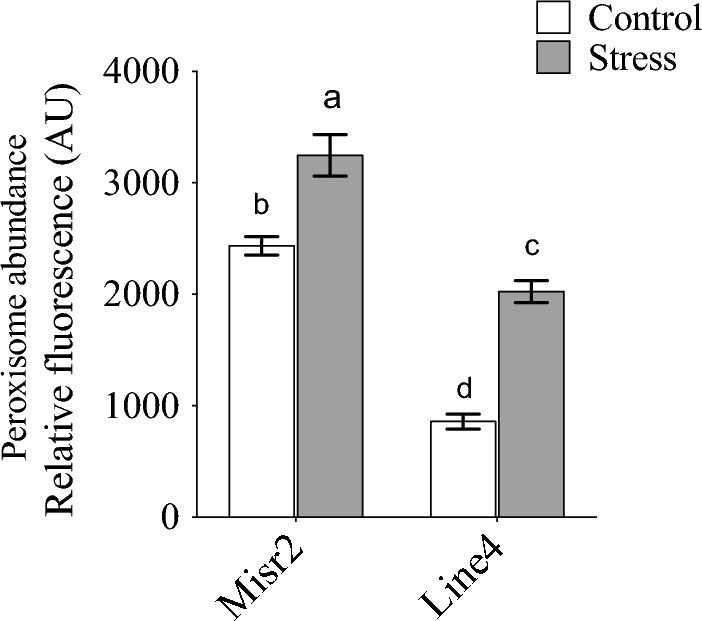
Average flag leaf peroxisome abundance of the tolerant (Misr2) and the susceptible (Line4) wheat genotypes grown under control and stress environments. Vertical bars with different lowercase letters above are significantly different between treatments (per genotype) at *P* = *0.05* by Tukey’s test, capped bars represent SD.

Regarding antioxidant enzyme activity, heat stress increased POX, SOD, and CAT activity in both genotypes (Fig. 5A–C). Although the genotypes did not differ significantly under stress, Misr2 showed a greater fold increase in POX and CAT relative to its control (Fig. 5A,C). SOD activity was notably higher in Misr2 following heat stress. At the same time, Line4 showed no significant change in the same parameter compared with its control (Fig. 5B). Heat stress also significantly increased peroxisome abundance in the flag leaves of both genotypes (Fig. 6). Misr2 maintained more than twice the peroxisome abundance of Line4 under control conditions and retained the highest levels under stress, demonstrating a greater inherent capacity to mitigate oxidative damage.

### Gene expression of stress-related genes

**Fig. 7:**
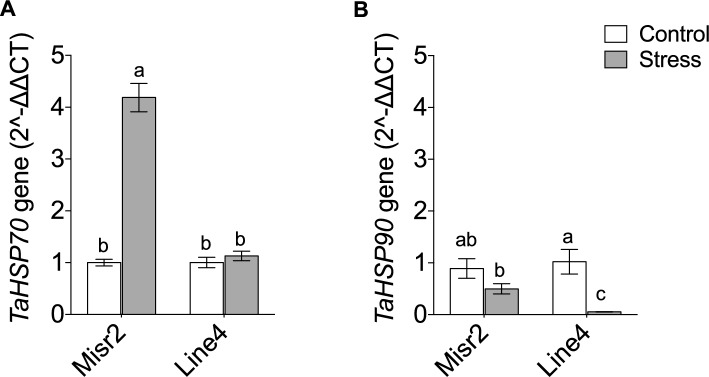
Expression profiling of (**A**) *TaHSP70* and (**B**) *TaHSP90* in the tolerant (Misr2) and the susceptible (Line4) wheat genotypes grown under control and stress environments. Vertical bars with different lowercase letters above are significantly different between treatments (per genotype) at *P* = *0.05* by Tukey’s test, capped bars represent SD.

Heat stress strongly upregulated *TaHSP70* in Misr2 by ~ 4.5-fold relative to the control. Still only minimally and non-significantly in Line4 (Fig. 7A). In contrast, heat stress significantly downregulated *TaHSP90* in Line4, whereas it did not significantly alter its expression in Misr2 (Fig. 7B).

**Fig. 8:**
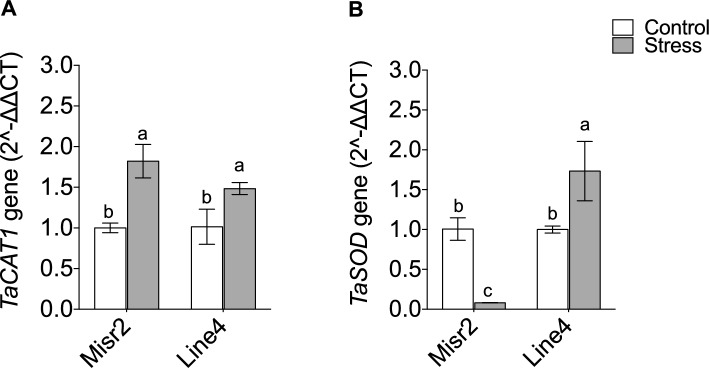
Expression profiling of catalase of (**A**) Catalase (*TaCAT1*) and (**B**) superoxide dismutase (*TaSOD*) in the tolerant (Misr2) and the susceptible (Line4) wheat genotypes grown under control and stress environments. Vertical bars with different lowercase letters above are significantly different between treatments (per genotype) at P = 0.05 by Tukey’s test, capped bars represent SD.

For antioxidant-related genes, heat stress upregulated *TaCAT1* in both genotypes without producing distinct expression patterns between them (Fig. 8A). In contrast, it downregulated *TaSOD* in Misr2 but upregulated it in Line4 relative to their respective controls (Fig. 8B).

**Fig. 9:**
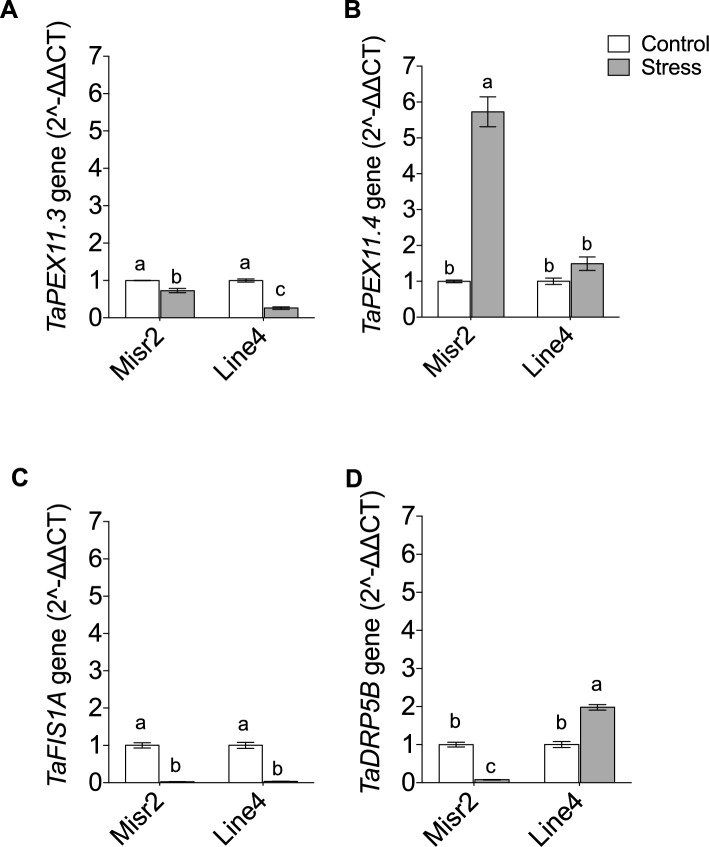
Expression profiling of peroxisome biogenesis genes (**A**) *TaPEX11.3*, (**B**) *TaPEX11.4*, (**C**) *TaFIS1A,* and (**D**) *TaDRP5B* in the tolerant (Misr2) and the susceptible (Line4) wheat genotypes grown under control and stress environments. Vertical bars with different lowercase letters above are significantly different between treatments (per genotype) at P = 0.05 by Tukey’s test, capped bars represent SD.

Heat stress also caused genotype-specific changes in peroxisome biogenesis genes by downregulating *TaPEX11.3* and *TaFIS1A* in both genotypes (Fig. 9A,C). However, it strongly upregulated *TaPEX11.4* (~ 5.5-fold) in Misr2, with no significant change in Line4 relative to their respective controls (Fig. 9B). Additionally, heat stress downregulated *TaDRP5B* in Misr2. Still, it upregulated it in Line4 (Fig. 9D). These transcriptional responses demonstrate that the two wheat genotypes regulate protein stability, oxidative stress defense, and organelle biogenesis differently under heat stress.

### Protein–protein interaction networks linked to heat stress response

**Fig. 10:**
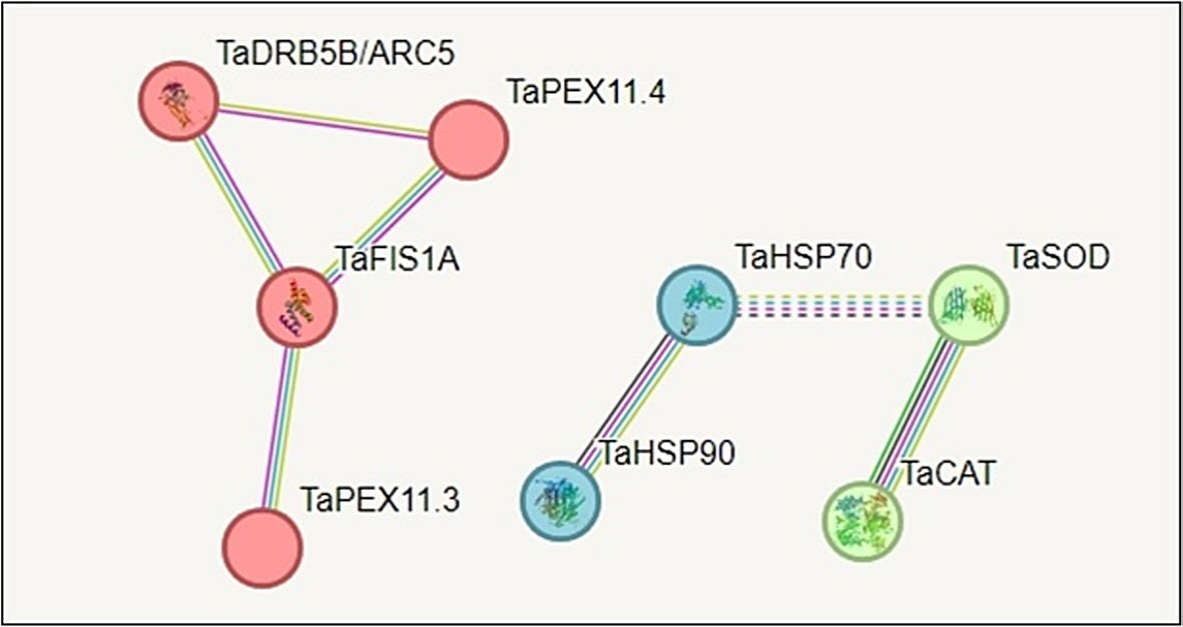
Predicted protein–protein interaction (PPI) model of heat shock proteins (HSP70, HSP90-1), antioxidant proteins (SOD, CAT), and peroxisomal biogenesis machinery proteins (PEX11.3, PEX11.4, FIS1A, DRP5B) using the protein sequences of wheat transcripts, the network developed by the STRING program (https://string-db.org/). Network edges: Line thickness indicates the degree of confidence in the data, supported by a P-value < 1.0e-16. The minimum protein–protein interaction (PPI) is at a medium confidence level of 0.700. Proteins were clustered into red (peroxisome network), blue (Heat shock protein network), and green (antioxidant protein network) colors using the K-means clustering approach.

The predicted protein–protein interaction (PPI) network among the eight encoded proteins (STRING database) showed medium confidence. The analysis did not detect direct interactions between heat shock, antioxidant, and peroxisome biogenesis proteins in wheat or their homologs. However, genes within each functional group exhibited internal co-expression relationships, and *TaHSP70* co-expressed with *TaSOD* (Fig. 10).

### Correlation and principal component analysis (PCA)

**Fig. 11:**
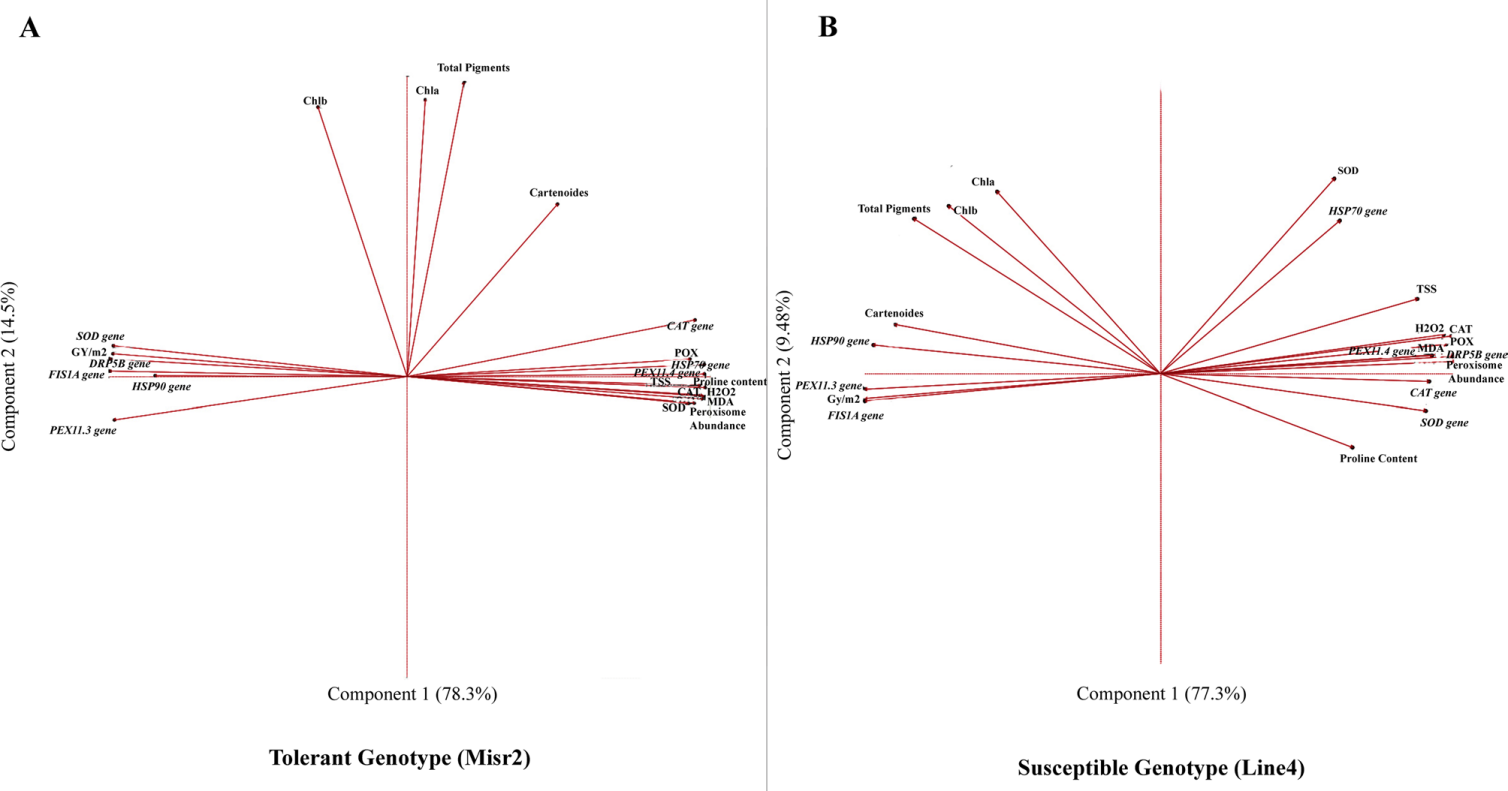
Principal components analysis (PCA), biplots obtained from physiological and gene expression data for (**A**) tolerant genotype (Misr2) and (**B**) susceptible genotype (Line4). The abbreviations refer to: *HSP gene* heat shock protein gene expression, *SOD gene* superoxide dismutase gene expression, *CAT gene* catalase gene expression, *PEX11.3 gene* peroxin11.3 gene expression, *PEX11.4 gene* peroxin11.4 gene expression, *FIS1A gene* mitochondrial fission1 gene expression, *DRP5B gene* dynamin-related protein 5B gene expression, *Chl* a chlorophyll a, *Chl* b chlorophyll b, *Car.* carotenoids, *TSS* total soluble sugars, *H*_*2*_*O*_*2*_ hydrogen peroxide, *MDA* malondialdehyde, *SOD* superoxide dismutase activity, *POX* peroxidase activity, *CAT* catalase activity, *GY/m*^*2*^ grain yield/m^2^.

Correlation and PCA analyses clarified wheat genotype-specific heat-stress-responsive strategies (Fig. 11; Table S1). In Misr2, ROS strongly correlated with MDA (r = 0.97), peroxisome abundance (r = 0.95), antioxidant enzymes (CAT, SOD, POX; r ≥ 0.96), proline, and TSS (r = 0.99 each). ROS also correlated positively with *TaHSP70* (r = 0.98), *TaPEX11.4* (r = 0.99), and *TaCAT1* (r = 0.92), but negatively with *TaHSP90*, *TaSOD*, *TaPEX11.3*, *TaFIS1A*, and *TaDRP5B* (r ≤ –0.83). In Line4, ROS-related traits clustered with osmoprotectants (MDA, TSS, proline), indicating a coordinated association between oxidative stress markers and osmoprotective responses that mitigate heat-induced cellular damage. PCA explained 93.1% of the variation in Misr2 and 86.1% in Line4 (Fig. 11; Table S1). Total soluble sugars and peroxisome abundance emerged as potential biomarkers for breeding heat-tolerant wheat genotypes.

### Differential gene network polarity defines genotypic heat stress response

**Fig. 12:**
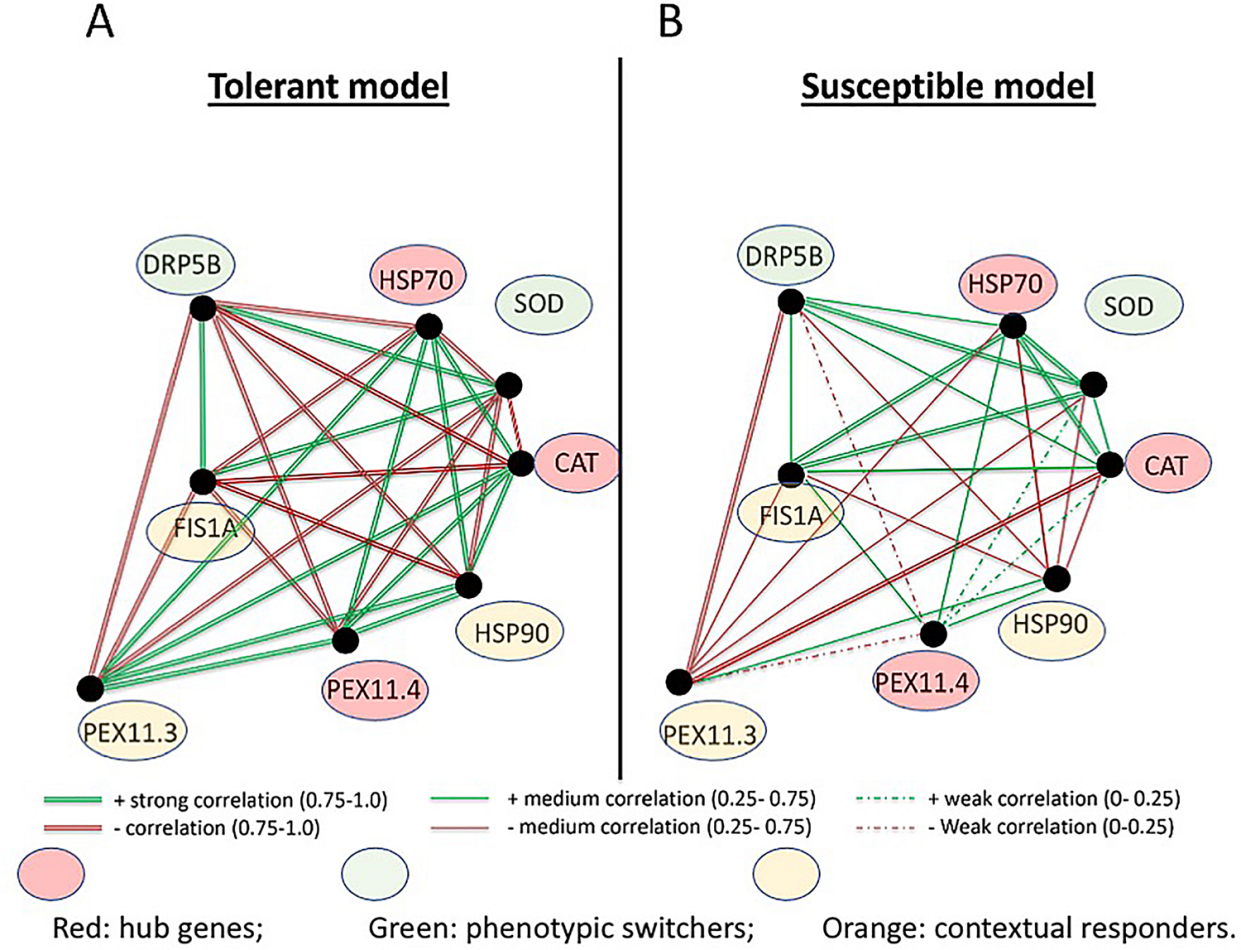
Correlation-based network model of heat shock proteins (*TaHSP70, TaHSP90*), antioxidant enzymes (*TaSOD, TaCAT*), and peroxisome biogenesis proteins (*TaPEX11.3, TaPEX11.4, TaFIS1A, TaDRP5B*) based on principal component analysis (PCA) loadings. Line styles represent correlation strength: double line = strong (r ≥ 0.75), solid line = medium (0.25 ≤ r < 0.75), dashed line = weak (r < 0.25). Colors indicate correlation direction (green = positive, red = negative). Node colors represent functional roles: red = hub genes, green = phenotypic switchers, orange = contextual responders. The models illustrate potential regulatory crosstalk in tolerant (**A**) and susceptible (**B**) genotypes.

The heat-tolerant wheat genotype, Misr2, formed a highly structured and polarized gene network (Fig. 12). In contrast, the susceptible genotype, Line4, displayed a fragmented architecture dominated by medium to weak correlations (Fig. 12). *TaHSP70*, *TaCAT1*, and *TaPEX11.4* exhibited the highest connectivity and the most significant shifts in correlation polarity between genotypes. Specifically, six of seven *TaCAT1-*associated correlations showed the same polarity (positive to negative or vice versa) when comparing the heat-tolerant genotype Misr2 with the susceptible genotype Line4. In contrast, *TaHSP70* and *TaPEX11.4* showed five and three such polarity reversals, respectively. In addition, *TaPEX11.4* showed weaker associations, with four correlations decreasing from strong to moderate strength, consistent with its central position and multiple network connections.

In contrast, *TaPEX11.3*, *TaFIS1A*, *TaDRP5B*, *TaHSP90*, and *TaSOD* maintained stable co-regulation among themselves but showed genotype-dependent changes in their associations with the three hub genes. Specifically, in the heat-tolerant genotype Misr2, *TaPEX11.3* showed strong correlations with *TaHSP70*, *TaCAT1*, and *TaPEX11.4*, whereas these correlations reversed in the susceptible genotype Line4. *TaFIS1A*, *TaDRP5B*, and *TaSOD* showed genotype-dependent sign reversals in their correlations with the hub genes. *TaHSP90* did not reverse correlation sign but instead shifted from strong with *TaPEX11.4* in Misr2 to a moderate correlation in Line4, consistent with overall network fragmentation in the susceptible genotype.

Gene expression analysis revealed the upregulation of *TaHSP70*, *TaCAT1*, and *TaPEX11.4* in the tolerant genotype, and downregulation of *TaPEX11.3*, *TaFIS1A*, *TaDRP5B*, *TaHSP90*, and *TaSOD* (Figs. 7–9)*.* Gene-to-trait correlations further linked these hub genes to phenotypic performance (Fig. 11). *TaHSP70*, *TaCAT1*, and *TaPEX11.4* displayed similar correlation patterns with grain yield, TSS, and proline in both genotypes, with stronger associations in Misr2, particularly for *TaHSP70* (Fig. 11A*)*. In contrast, *TaSOD* and *TaDRP5B* reversed their correlations with all measured traits, including yield (Fig. 11), between tolerant and susceptible genotypes.

These coordinated expression and correlation patterns were integrated into genotype-specific regulatory network models, revealing a highly structured network in Misr2 and a fragmented architecture in Line4 (Fig. 12).

## Discussion

This study reveals distinct regulatory mechanisms of thermotolerance in wheat by comparing heat-tolerant and heat-susceptible genotypes under field-induced heat stress. We address a key knowledge gap on how heat-stress signaling pathways, osmoprotectants, and coordinated phenotypic–genotypic defenses interact. Our results identify a previously unreported crosstalk among heat shock proteins (*TaHSP70, TaHSP90*), antioxidant enzymes (*TaCAT1, TaSOD*), and peroxisome biogenesis genes (*TaPEX11.3, TaPEX11.4, TaFIS1A, TaDRP5B*) absent from current PPI databases. Disruption of this network’s correlations appears to underlie reduced heat tolerance in the studied wheat genotypes, Misr2 (heat-tolerant) and Line 4 (heat-susceptible).

### Differential mechanisms of heat stress response

**Fig. 13:**
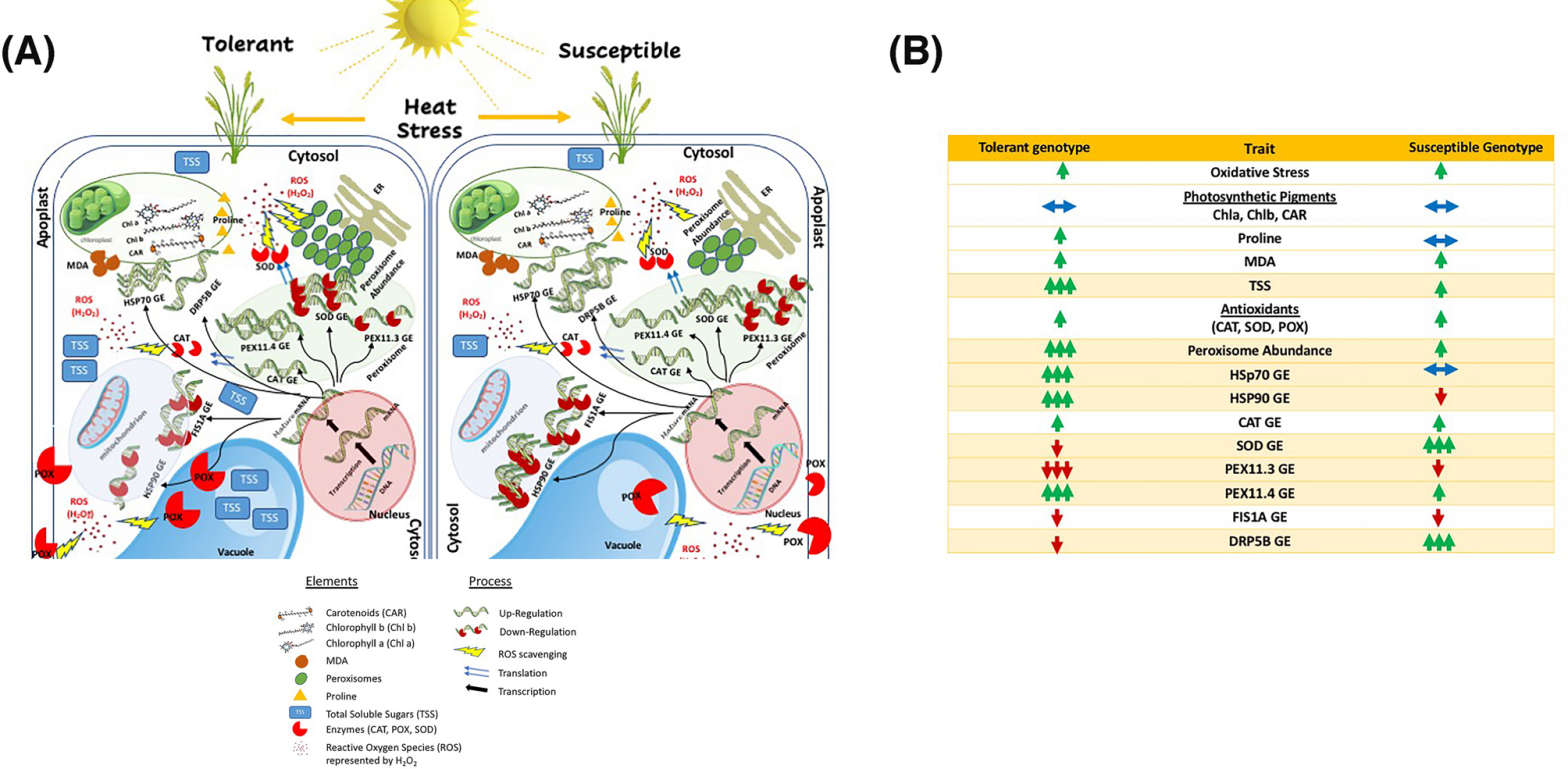
Integrated heat stress response in tolerant (Misr2) and susceptible (Line4) wheat genotypes. (**A**) Mechanistic model illustrating genotype-specific responses under heat stress. The schematic highlights coordinated regulation among reactive oxygen species (ROS, shown as H₂O₂), antioxidant enzymes (CAT, POX, SOD), osmoprotectants (MDA, TSS, proline), peroxisome biogenesis genes (*TaPEX11.3****,**** TaPEX11.4, TaFIS1A, TaDRP5B*), and heat shock proteins (*TaHSP70, TaHSP90*). The tolerant genotype exhibits stronger transcriptional activation, enhanced protein stability, and more effective ROS detoxification, contributing to cellular protection and photosynthetic pigment stability. (**B**) Comparative summary of physiological and molecular responses to heat stress. Green upward arrows indicate increases or upregulation; red downward arrows indicate decreases or downregulation; blue double-headed arrows indicate no significant change. All responses are relative to non-stressed controls. Quantitative data support this schematic, capturing trait-level divergence between genotypes. The legend explains the molecular elements and regulatory processes shown in the diagram.

A comparative schematic representation (Fig. 13) summarizes the physiological, biochemical, and transcriptional changes of wheat to heat stress. Both genotypes showed elevated oxidative stress (H₂O₂) under heat stress. Notably, the heat-tolerant Misr2 mounted a more coordinated response, accumulating higher total soluble sugars and proline—enhancing osmotic adjustment and stabilizing proteins and membranes. Antioxidant enzyme activities (CAT, POX, SOD) increased in both genotypes but aligned more closely with peroxisome abundance in the heat-tolerant genotype.

Gene expression analysis revealed a strong upregulation of *TaHSP70, TaPEX11.4*, and *TaCAT1* in the heat-tolerant genotype, and a downregulation of *TaSOD, TaPEX11.3*, *TaFIS1A,* and *TaDRP5B*, with only a slight upregulation of *TaHSP90*, reflecting balanced ROS detoxification and peroxisomal proliferation. In contrast, the heat-susceptible genotype upregulated *TaCAT1, TaSOD,* and *TaDRP5B*. It downregulated *TaHSP90, TaPEX11.3*, and *TaFIS1A,* with minimal change in *TaPEX11.4,* suggesting activation of antioxidant and peroxisome division–related stress responses, but limited coordination with HSP-mediated protective mechanisms. Despite the increase in peroxisome abundance in response to heat stress, the integration of this response with antioxidant and osmoprotective mechanisms appeared less effective than in the heat-tolerant genotype. Photosynthetic pigments (Chl a, Chl b, and carotenoids) remained stable in both genotypes, indicating stable photosystems under heat stress. Our findings are consistent with^12^, who reported that heat-tolerant wheat genotypes accumulate higher levels of heat-tolerance–associated biochemical markers, such as proline and total soluble sugars, and exhibit greater relative fold expression of stress-responsive genes compared to heat-sensitive genotypes. In their study, heat-sensitive genotypes also showed a more pronounced decline in grain yield than tolerant genotypes under late sowing conditions^12^.

### Phenotype–genotype associations

#### Peroxisome biogenesis machinery network

Peroxisomes are central to plant stress adaptation by regulating ROS scavenging and maintaining cellular homeostasis^26,33,34^. Advances in high-throughput peroxisome quantification^29–31,35^ have established peroxisome abundance as a phenotypic biomarker for abiotic stress tolerance. Under heat stress, both wheat genotypes increased peroxisome numbers; however, the heat-tolerant genotype maintained a higher absolute abundance, whereas the susceptible genotype showed a greater relative increase than its control. This pattern is consistent with stress-induced peroxisome proliferation reported under elevated oxidative load or increased cellular stress demand**,** rather than constitutive peroxisome maintenance^25,26,33^.

Grain yield correlations revealed that in the susceptible genotype, increased peroxisome abundance was negatively associated with productivity, suggesting a stress-induced, compensatory response that may divert metabolic resources away from growth and yield formation. In contrast, the heat-tolerant genotype showed positive correlations between peroxisome abundance and protective traits, such as TSS and proline, suggesting a more coordinated integration of peroxisome dynamics with osmoprotective and oxidative-stress-mitigation mechanisms. Similar associations between stress-induced peroxisome proliferation, oxidative load, and growth or yield trade-offs have been reported in plants under abiotic stress^8,25,26,33^.

At the transcriptional level, *TaPEX11.4* induction paralleled peroxisome accumulation in both genotypes, but *TaPEX11.3* was notably downregulated in the heat-susceptible genotype, suggesting functional decoupling or temporal shifts in isoform contribution. *TaFIS1A*, an organelle fission factor, was also downregulated under heat stress in both genotypes. Across most traits, *TaFIS1A* showed negative correlations, but it was positively associated with grain yield and Chl b, suggesting a potential link to pigment maintenance rather than broad stress protection. In the susceptible genotype, *TaFIS1A* also correlated positively with carotenoids, indicating a genotype-specific connection to photoprotective pigment dynamics that may not directly reflect peroxisome proliferation efficiency. **S**imilar isoform-specific regulation of PEX genes, stress-dependent modulation of organelle fission factors, and functional coupling between redox metabolism and photosynthetic pigment maintenance have been reported under abiotic stress conditions^8,25,36,37^.

The heat-tolerant wheat genotype appears to rely on a high constitutive peroxisome pool, with targeted induction of *TaPEX11.4* under heat stress. By contrast, the susceptible genotype strongly upregulated *TaDRP5B*, a gene encoding a dynamin-related protein required for chloroplast division^38,39^. Together with its negative correlation with carotenoids, this pattern is more consistent with chloroplast remodeling and photoprotective adjustment rather than enhanced peroxisome division^8,40^.

#### Antioxidant enzyme network

Heat stress increased H₂O₂ and MDA levels in both genotypes, indicating increased oxidative stress. H₂O₂ functions as both a damaging agent and a redox signaling molecule, whereas MDA represents a lipid peroxidation–derived reactive carbonyl species associated with oxidative damage and potential secondary regulatory effects^41,42^. The tolerant genotype accumulated higher TSS and proline, both of which correlated positively with peroxisome abundance, suggesting enhanced osmotic adjustment alongside ROS management.

Antioxidant enzymes (SOD, CAT, POX) were active in ROS scavenging in both genotypes, but their regulation differed. The heat-tolerant genotype exhibited higher SOD activity despite lower *TaSOD* transcript levels, suggesting post-transcriptional regulation via mRNA stability or protein turnover^43^. CAT activity was stable across genotypes, reflecting its constitutive role at the evaluated wheat growth stage. At the same time, POX contributes to fine-tuning cellular H₂O₂ levels and thereby indirectly influences ROS-mediated signaling processes, in addition to its scavenging function^44^.

Gene–phenotype associations further highlighted genotype-specific strategies in heat-adaptative responses of wheat. Both genotypes upregulated *TaCAT1* under heat stress, confirming its central role in ROS detoxification^45^. In the susceptible genotype, *TaCAT1* and *TaSOD* expression correlated negatively with grain yield and photosynthetic pigments but positively with oxidative damage markers (H₂O₂, MDA) and osmolytes**,** suggesting a reactive, damage-driven antioxidant response typically associated with elevated oxidative burden rather than effective stress tolerance^8,46^. In the heat-tolerant genotype, *TaCAT1* maintained positive correlations with most traits, whereas *TaSOD* showed the opposite pattern, correlating negatively with all measured traits except yield. This uncoupling between transcript abundance and enzyme activity supports the contribution of post-transcriptional regulatory mechanisms in sustaining thermotolerance**,** as widely reported under heat and other abiotic stresses^43,47^.

#### Heat shock protein network

Heat shock proteins safeguard cellular structures and modulate stress signaling. In plants, HSP70 family members primarily function as cytoprotective chaperones that maintain proteostasis under heat stress**,** whereas HSP90 is more closely associated with the regulation of stress signaling components, including heat shock transcription factors and kinase cascades^48–50^. In the heat-tolerant genotype, elevated *TaHSP70* expression coupled with reduced *TaHSP90* expression suggests a functional shift toward enhanced protein protection at the anthesis stage^17^. In both genotypes, *TaHSP70* showed a positive correlation with H₂O₂, consistent with ROS-mediated activation of HSP expression, a well-established mechanism linking oxidative signaling to heat shock responses^51^.

Photosynthetic pigments, including chlorophylls and carotenoids, remained stable under heat stress in both genotypes, likely reflecting long-term acclimation under field conditions rather than acute stress responses^52,53^. Carotenoids serve as key antioxidants, protecting the photosynthetic machinery during light harvesting and dissipating excess excitation energy^54,55^. Notably, despite unchanged carotenoid content, grain yield correlations with carotenoids diverged sharply between genotypes: positive in the heat-tolerant genotype and negative in the susceptible genotype**.** This contrast suggests genotype-specific differences in how carotenoid-associated photoprotective capacity relates to productivity under heat stress, rather than changes in carotenoid content itself.

Network-level correlations in the heat-tolerant genotype revealed a positive association between carotenoids and *TaHSP70*, indicating coordinated regulation of pigment- and chaperone-related traits within the stress-response network. In contrast, this association was negative in the susceptible genotype, suggesting genotype-specific differences in the relationship between carotenoid status and HSP70 expression under heat stress. Both *TaHSP70* and carotenoids are known to contribute to cellular stress mitigation by stabilizing proteins and protecting against oxidative damage^48,49,55^. *TaDRP5B* expression was negatively correlated with carotenoids in both genotypes, potentially reflecting altered chloroplast dynamics associated with stress responses rather than direct effects on pigment stability^38,39^.

### Crosstalk between the three networks

The three molecular networks—peroxisome biogenesis, antioxidant defense, and heat shock proteins—exhibited distinct yet interconnected regulation under heat stress. In the heat-tolerant genotype, peroxisome abundance correlated positively with *TaCAT1* and *TaHSP70* but negatively with *TaSOD* and *TaHSP90*, consistent with a coordinated transition from stress perception toward balanced ROS detoxification and proteostatic maintenance. Carotenoids and *TaHSP70* formed a cooperative module, while peroxisomes and *TaCAT1* anchored the antioxidant–organelle interface.

In the susceptible genotype, network integration was weaker and less coordinated: peroxisome proliferation coincided with higher levels of oxidative damage markers, and co-regulation of *TaCAT1* and *TaSOD* was more closely associated with oxidative stress indicators than with yield stability. Associations between carotenoids and stress-related traits also differed from those observed in the tolerant genotype, suggesting altered coupling between photoprotective components and chaperone responses.

Correlation polarity shifts between networks, particularly for *TaSOD*, *TaDRP5B*, and carotenoids, highlight genotype-specific rewiring of stress-response modules. Together, this integrated phenotype–genotype framework indicates that thermotolerance in wheat depends not only on the capacity of individual protective networks but also on their ability to coordinate dynamically under a defined heat stress condition.

### Modular gene network reorganization supports wheat thermotolerance

This study reveals a distinct transcriptional coordination pattern underlying genotype-specific heat stress responses in wheat, characterized by a modular network architecture with dynamic hub genes and context-sensitive peripheral modules. Correlation-based network analysis identified *TaHSP70, TaCAT1,* and *TaPEX11.4* as central hub genes that exhibited extensive polarity shifts in their gene–gene interactions between heat-tolerant and heat-susceptible wheat genotypes. These genes were also upregulated in the tolerant background, supporting their involvement in genotype-specific stress regulation. In contrast, *TaPEX11.3, TaFIS1A, TaDRP5B, TaHSP90, and TaSOD* maintained consistent internal co-regulation but showed reversed correlations with the hub genes across genotypes. These connectivity changes suggest altered regulatory alignment between antioxidant and peroxisomal pathways under heat stress.

A key observation was the divergence in gene-to-phenotype correlations. While *TaHSP70, TaCAT1, and TaPEX11.4* maintained consistent correlation directions with key phenotypic traits in both genotypes, their associations were stronger in the tolerant genotype, particularly for *TaHSP70*. In contrast, *TaSOD* and *TaDRP5B* exhibited fully reversed correlations across all evaluated traits, including grain yield. These two genes, therefore, represent potential phenotype-level switching nodes, whose contributions to stress performance may shift depending on genotype context, consistent with network-based models of stress-response plasticity and context-dependent gene function^56,57^.

By comparison, *TaPEX11.3, TaFIS1A,* and *TaHSP90* showed more limited polarity shifts and weaker or conserved trait correlations, suggesting that their roles may be more context-dependent within the broader stress-response network. Together, these findings support a modular view of transcriptional regulation under heat stress, in which genotype-specific rewiring of hub gene connectivity and peripheral gene–trait relationships contribute to phenotypic differences in stress adaptation. To our knowledge, this is the first report to link correlation polarity switching at both transcriptional and phenotypic levels with heat tolerance in wheat, providing a conceptual framework for future functional validation and stress-resilient crop improvement strategies.

## Conclusions

This study integrates physiological traits, gene expression profiles, and grain yield data to elucidate mechanisms underlying heat tolerance in two contrasting wheat genotypes. The heat-tolerant genotype, Misr2, possessed better coordination of responses related to antioxidants, peroxisomes, and HSPs than the heat-sensitive wheat line, Line4. Misr2 accumulated a higher amount of total soluble sugar (TSS) and transcription of *TaPEX11.4* and *TaHSP7*0 than Line4, which reflects greater metabolic and proteostatic protection in response to heat stress.

There was an increase in the levels of MDA in heat-stressed plants of all genotypes, while the response of proline was genotype-dependent, rather than being the same in all genotypes. There was also an induction of *TaCAT1* in heat-stressed Misr2 and Line4, suggesting the constitutive role of the enzyme in the antioxidant system rather than genotype-related expression. There was no significant difference in the expression of *TaCAT1* between the genotypes, suggesting that heat tolerance can be a function of regulatory systems rather than the expression of the enzyme itself.

The difference in expression patterns between *TaHSP70* and *TaHSP90* in Misr2 is in line with functional differentiation between the promotion of chaperone-mediated protein protection and modifications in stress-associated signaling components regulation, not requiring temporal relationships based on single time point sampling. The disparity between *TaSOD* transcripts expression and SOD activity reinforces the view that post-transcriptional regulation exists as an adaptation for thermotolerance.

Gene-trait network analysis showed that thermotolerance has a module structure, in which *TaHSP70*, *TaCAT*1, and *TaPEX11.4* are hubs showing a genotype-dependent reversal of correlation polarity, and *TaSOD* and *TaDRP5B* represent phenotype-switching nodes. The positive correlations between yield-related traits and *TaPEX11.4, TaHSP70, TaCAT1*, and peroxisome density indicate their potential use as biomarkers for identifying heat-tolerant wheat lines, in terms of network behavior. Although further research is needed to confirm these results in other wheat genetic lines and environments, a functional approach to combine physiological and genetic traits for wheat improvement at increased temperatures is provided here.

## Materials and methods

### Plant material and stress treatments

Two contrasting spring bread wheat (*Triticum aestivum* L.) genotypes were used: Misr2 (SKAUZ/BAV92), a thermotolerant cultivar, and Line4 (KINGBIRD#1//INQALAB91*2/TUKURU), a thermosusceptible line. Misr2 seeds were obtained from the Agriculture Research Center, Giza, Egypt, while Line4 seeds were imported from CIMMYT, Mexico. Both genotypes were selected from a panel of 20 wheat genotypes based on evaluation trials conducted over two successive seasons using late sowing procedures^3^. The study re-evaluated two genotypes (Misr2 and Line4) under two sowing conditions: the expected sowing date (November 28th) and a late sowing date (January 24th). Heat stress was imposed by delaying the sowing date by 57 days beyond the normal planting time, a widely used field-based approach for inducing terminal heat stress in wheat^2,58^. Sampling occurred during anthesis. Figure S1 presents the average seasonal minimum and maximum temperatures for both conditions, as well as the temperatures at sampling time. On the day of sampling, the maximum temperatures were about 26.6 and 33.5 °C, for regular and late sowing, respectively. Both genotypes were cultivated in the field within 1.0 × 1.0 m^2^ plots with a row-to-row spacing of 25 cm for each sowing date at the Cairo University Research Farm, Giza, Egypt, in clay-loam soil characterized by the following physicochemical properties: sand (33.1%), silt (34.6%), clay (32.3%), pH (7.49), and electrical conductivity (E.C., 1.58 dS/m). Surface supplementary irrigation was applied as needed throughout the wheat growth period. All agricultural practices followed standard wheat-growing protocols. The Egyptian Meteorological Authority (EMA) recorded maximum and minimum temperatures at regular intervals during the 2019/2020 growing season.

### Sampling protocol

The flag leaves from internal rows were collected from both genotypes at the 7^th^ day after anthesis (DAA) in triplicate for each genotype and treatment. Samples were immediately frozen in liquid nitrogen and stored at − 80 °C until further use. Sampling was performed at 1:00 PM, when solar radiation was perpendicular to the surface, to minimize temperature variations. At the end of the season, all plants were harvested, and grain yield per square meter was measured for both genotypes and treatments.

### Phenological and biochemical parameters

The endogenous content of H_2_O_2_ was measured following the method of Velikova et al.^59^. Chlorophyll a (Chl a) and b (Chl b) and carotenoids (CAR) in wheat leaves were calculated according to Lichtenthaler and Buschmann^60^. Proline content was determined using the ninhydrin method as described by Bates et al.^61^. Total soluble sugars (TSS) were quantified using a glucose calibration curve, as described by DuBois et al.^62^. Malondialdehyde (MDA) content, a proxy for lipid peroxidation, was assessed according to the protocol of Chen and Wang^63^.

### Enzymatic measurements

Superoxide dismutase (SOD, EC 1.12.1.1) and catalase (CAT, EC 1.11.1.6) were extracted from leaf tissues, and their activities were quantified using a Cary Series UV–Vis spectrophotometer (Agilent Technologies) according to the method of Chen and Wang^63^. Peroxidase (POX, EC 1.11.1.7) enzyme was quantified according to the process of Kumar and Khan^64^.

### Measuring peroxisome abundance

The total protein content was extracted from each biological replicate per genotype and treatment. For each biological replicate, peroxisome abundance was assayed in three technical repeats. The fluorescent probe N-BODIPY was used to detect peroxisome abundance in the protein extracts. Extraction and quantification were performed according to the protocol described by Fahy et al.^29^, using a CLARIOstar. Spectrofluorometer with excitation and emission wavelengths set at 490 and 530 nm, respectively.

### Gene expression profiling using quantitative real-time PCR (qRT-PCR)

Total RNA was extracted from wheat leaves using the GeneJET™ Plant RNA Purification Mini Kit (ThermoFisher), following the manufacturer’s instructions. RNA quality and quantity were evaluated using a NanoDrop™ One/OneC Microvolume UV–Vis Spectrophotometer (ThermoFisher), and concentrations were adjusted to 50 ng/μL. First-strand cDNA was synthesized using the High-Capacity cDNA Reverse Transcription Kit (Applied Biosystems). Quantitative PCR (qPCR) was performed using PowerUp™ SYBR™ Green Master Mix (Thermo Fisher) in 20 μL reactions, with three biological and three technical replicates per sample.

Primers for the target genes, including the wheat housekeeping gene *Actin-7*^65^ (GenBank accession XM_044594643.1), were designed using Primer-BLAST software (NCBI), which integrates Primer3 (version 2.5.0) for primer design and employs BLAST and global alignment algorithms to screen primer pairs against the wheat genome and transcriptome databases (Table 1). This process ensured specificity by avoiding primer pairs with potential cross-homology to non-target gene transcripts. The designed primers were synthesized using the Invitrogen Synthesis Facilities Service (Town, Country).

**Table 1:** A list of target genes, specific primers used for the quantitative real-time PCR (qRT-PCR).

Genome location	Transcripts	Accession number	Primer sequences (5’-3’)	Primer	Gene
**1D** **1B** **1A**	TraesCS1A02G285000 TraesCS1B02G294300 TraesCS1D02G284000	XM_044594775.1	CTTCGTCCAGGAGTTCAAGC	Forward	HSP70
			GTCGATCTCGATGGTGGTTT	Reverse	
**2A** **2B** **2D**	TraesCS2A02G033700 TraesCS2B02G047400 TraesCS2D02G033200	XM_044598798.1	CGAGTACGGGTGGACGGCCAACAT	Forward	HSP90
			TCTCGAAGAGCAGCATCACGAGGT	Reverse	
**7A** **7B** **7D**	TraesCS7A02G292100 TraesCS7B02G197300 TraesCS7D02G290700	XM_044587054.1	TCCTTTGACTGGCCCTAATG	Forward	SOD/SOD(Cu–Zn)
			CTTCCACCAGCATTTCCAGT	Reverse	
**5A** **4B** **4D**	TraesCS5A02G498000 TraesCS4B02G325800 TraesCS4D02G322700	NM_001405704.1	CAAGAGCGATTCATCAACAGAT	Forward	CAT1
			AGACCAGTAGGAGAGCCAGATG	Reverse	
**4A** **4B** **4D**	TraesCS4A02G117800 TraesCS4B02G186600TraesCS4D02G187900	XM_044519518.1	CGC TAG GGG ACG TGA CTA A	Forward	PEX11.3
			CAG CGC CGA CAG CAA TC	Reverse	
**2A** **2B** **2D**	TraesCS2A02G368300 TraesCS2B02G385400 TraesCS2D02G365100	XM_044469291.1	CAA CCC GTT CTG CAA CCA C	Forward	PEX11.4
			TTC CTA TAC CAC CCA GCC CA	Reverse	
**1A** **1B** **1D**	TraesCS1A02G239800 TraesCS1B02G252000 TraesCS1D02G240000	XM_044550628.1	TCCAAGCAGACTGATGATGTG	Forward	FIS1A
			TGGGCTGGTGGTTTTATCAAGA	Reverse	
**5A** **5B** **5D**	TraesCS5A02G114600 TraesCS5B02G120600 TraesCS5D02G124800	XM_044524285.1	AGGGAGGAAATAGTCAACGCC	Forward	DRP5B
			TCAAATGTGTCGCGTGCTTT	Reverse	
		XM_044594643.1	GGTCCAAACGAAGGATAGCA	Forward	Actin -7
			GCGGTCGAACAACTGGTATT	Reverse	

*HSP* heat shock protein, *SOD* superoxide dismutase, *CAT* catalase, *PEX* Peroxin, *FIS* mitochondrial Fission1, *DRP* dynamin-related protein.

The qPCR thermal cycling conditions followed the manufacturer’s recommendations for the PowerUp™ SYBR™ Green Master Mix on an Applied Biosystems 7500 Fast Real-Time PCR System. The protocol consisted of four stages: an initial uracil-DNA glycosylase (UDG) activation step at 50 °C for 2 min, followed by Dual-Lock™ DNA polymerase activation at 95 °C for 2 min. This step was followed by 40 cycles of denaturation at 95 °C for 3 s and annealing/extension at 60 °C for 30 s. A melt curve analysis was conducted at the end of the amplification cycles to verify the specificity of the PCR products.

Gene expression levels were quantified by calculating fold changes using the 2^(−ΔΔCt) method ± standard deviation (SD) across biological replicates, as described by Livak and Schmittgen^66^.

### Traits analysis strategy

Means and standard deviations were calculated for all measured traits to summarize variation across the two wheat genotypes, Misr2 and Line4, under contrasting sowing conditions. Throughout the manuscript, “control” refers to plants grown under normal sowing dates, and “stress” refers to those exposed to heat stress induced by late sowing.

A two-way ANOVA was then performed in JMP Pro (v8.0; SAS Institute, Cary, NC, USA) to estimate least-squares means (LS means), which adjust for the variance structure and potential data imbalance. Post-hoc comparisons were performed using Tukey’s honest significant difference (HSD) test to determine statistically significant differences among genotype X treatment combinations.

To identify trait–gene expression patterns, principal component analysis (PCA) was performed by integrating nine phenotypic traits with the expression profiles of eight heat-responsive genes. This multivariate approach enabled dimensionality reduction, facilitated genotype/treatment clustering, and revealed coordinated stress-response patterns in Misr2 and Line4. Means and standard deviations for all physiological traits and gene expression data were plotted using GraphPad Prism 8.0 (GraphPad Software, San Diego, CA, USA).

### Network construction approach

Genotype-specific gene co-expression networks were constructed by calculating pairwise Pearson correlation coefficients among the expression values of eight target genes: heat shock protein 70 (*TaHSP70*), heat shock protein 90 (*TaHSP90*), superoxide dismutase (*TaSOD*), catalase 1 (*TaCAT1*), fission protein 1A (*TaFIS1A*), dynamin-related protein 5B (*TaDRP5B*), peroxisomal biogenesis factor 11.3 (*TaPEX11.3*), and peroxisomal biogenesis factor 11.4 (*TaPEX11.4*). Hereafter, gene symbols are used throughout the manuscript.

The correlation coefficients were classified into strength-based categories to facilitate visual clarity (Fig. 12, see the legend for details). These categories were then used to construct genotype-specific co-expression networks, enabling both visual and quantitative comparison of transcriptional coordination under heat stress.

### Protein–protein interaction prediction

The encoded proteins of the studied genes were used as input for protein–protein interaction (PPI) prediction using the STRING database (version 12.0; https://string-db.org)^67^. *Triticum aestivum* was selected as the target organism. The analysis included interaction sources such as experimental data, curated databases, gene co-expression, gene neighborhood, gene fusion, and text mining. A minimum interaction confidence score of medium (≥ 0.400) was applied to filter interactions. The resulting PPI network was visualized to identify potential functional clusters and key interacting proteins. Gene ontology (GO) and KEGG^68,69^ pathway enrichment analyses were also performed within STRING to investigate the biological significance of the interactions.

## Supplementary Information

Supplementary Information 1.

Supplementary Information 2.

Supplementary Information 3.

## Data Availability

The datasets generated during the current study are publicly available in the NCBI Gene Expression Omnibus (GEO) repository under accession number **GSE326218** (Real-time quantitative PCR analysis of *Triticum aestivum* leaves under heat stress). The dataset includes the following samples: **GSM9625389** (Misr 2, non-stressed), **GSM9625390** (Misr 2, stressed), **GSM9625391** (Line 4, non-stressed), and **GSM9625392** (Line 4, stressed). Additional data supporting the findings of this study are included within the article and its Supplementary Information files.
