# Supplementary material for: Crosstalk of heat shock proteins and antioxidants with peroxisome biogenesis supports wheat thermotolerance
Source: Sci Rep. 2026 May 10;16:14700. doi: 10.1038/s41598-026-48451-0 (PMC13158294; doi:10.1038/s41598-026-48451-0)
Supplement: Supplementary file 2 — Supplementary Information 2. [file 41598_2026_48451_MOESM2_ESM.docx]

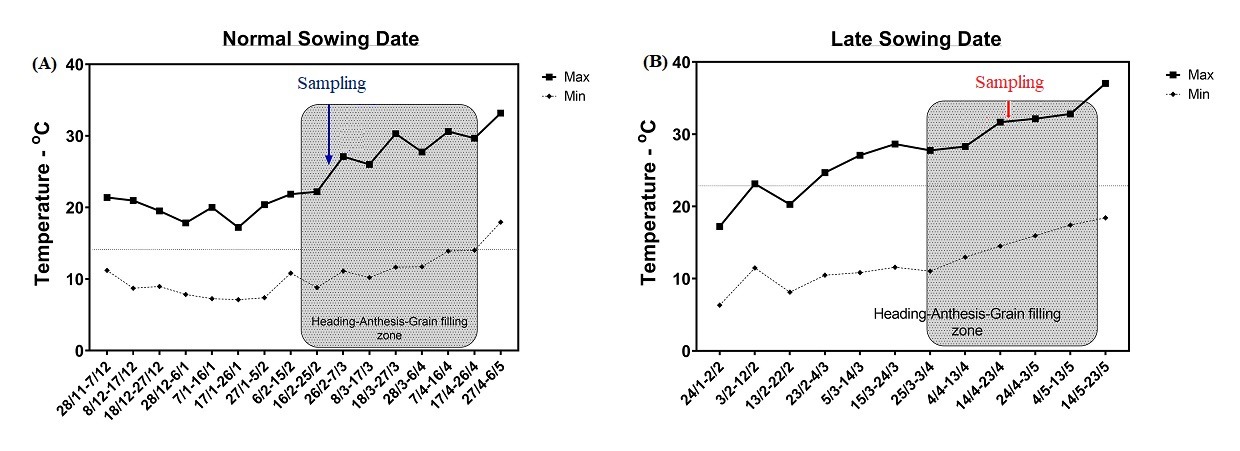


**Supplementary Fig. (S1):** Maximum and minimum air temperatures recorded during the 2019–2020 wheat-growing season, divided into ten-day intervals per month, based on data from the Egyptian Meteorological Authority (EMA). Panel (A) represents the normal sowing date, with sampling conducted at an average temperature of approximately 26.6°C. Panel (B) illustrates the late sowing date, reflecting heat-stress conditions, with sampling conducted at an average temperature of around 33.5°C. The shaded area in both panels highlights the heading–anthesis–grain filling zone.

**Supplementary Table (S1):** Assigned correlation values from Principal Component Analysis (PCA) for key traits in the tolerant genotype (Misr2, top) and the susceptible genotype (Line4, bottom) under heat stress conditions. The abbreviations refer to: HSP gene= heat shock protein gene expression, SOD gene= superoxide dismutase gene expression, CAT gene= catalase gene expression, PEX11.3 gene = peroxin11.3 gene expression, PEX11.4 gene = peroxin11.4 gene expression, FIS1A gene= mitochondrial fission1 gene expression, DRP5B gene= dynamin-related protein 5B gene expression, *Chl* a: chlorophyll a, *Chl* b: chlorophyll b, Car.: carotenoids, TSS: total soluble sugars, H_2_O_2_= hydrogen peroxide, MDA= malondialdehyde, SOD= superoxide dismutase activity, POX = peroxidase activity, CAT= catalase activity, GY/m^2^=grain yield/m^2^.

(Data is in a separate Excel file)

**Supplementary Table (S2):** Raw data for the relative gene expression levels of eight studied genes (*TaHSP70, TaHSP90, TaSOD, TaCAT1*, *TaPEX11.3, TaPEX11.4, TaFIS1A*, and *TaDRP5B*) in wheat genotypes Misr2 (tolerant) and Line4 (susceptible) under heat stress conditions. Expression levels are normalized to the housekeeping gene *TaActin7* and presented as means ± standard deviation from three biological replicates. The table shows genotype-specific expression data for heat shock proteins, antioxidant enzymes, and peroxisome biogenesis-related genes under heat stress. (Data is in a separate Excel file)
